# Point-of-Care Information in Open Access: A Time to Sow?

**DOI:** 10.1371/journal.pmed.1001870

**Published:** 2015-08-25

**Authors:** The PLOS Medicine Editors

## Abstract

The *PLOS Medicine* Editors support expanding the global benefits of clinical point-of-care information through open access and note challenges that may arise along the way.

Taking care of patients requires access to recent and reliable information. In high-income settings, what once involved thumbing through the few well-worn reference books that were close at hand (and, when that failed, paying a quick visit to the stacks in the nearest medical library) has become a matter of typing a search term or two and selecting the chapter of interest in an online, point-of-care reference. The change reflects a matter greater than simply convenience: with an ever-unfolding evidence base, with ever-increasing specialization and complexity, access to the latest information is an essential part of modern medicine. Surely it is well worth paying for?

Indeed, point-of-care references, such as DynaMed and UpToDate, are paid for, where individuals and institutions can afford them. But such resources are far from universally available, as James Heilman points out in an essay published in *PLOS Medicine* this week [1]. Heilman, an emergency room physician who has been editing medical content on Wikipedia since 2007 [2], asks why no high-quality, open access, point-of-care resource exists and considers what it would take to establish one. Medical journals, he concludes, could play a key role in generating high-quality content by publishing more peer-reviewed clinical review articles under an open copyright license.

The idea of collaboratively growing an open access, point-of-care reference has much to recommend it. Publication of the “seed” clinical reviews as peer-reviewed articles in respected journals could provide incentives for expert faculty to prioritize writing them because such widely beneficial work would likely gain recognition among academic institutions that value scholarship as community service. The journal articles’ open copyright license would permit subsequent adaptation of these reviews to “living documents” edited by communities of informed contributors, perhaps along the lines of the Wikipedia model, independently of the original journal. Community contributors could incorporate a wider range of treatment options than the original journal-based review might include, supporting greater choice in shared decision-making and personalized medicine. Open copyright licenses on these “living documents,” in turn, would facilitate universal access and reuse, without a need to negotiate fees or permissions.

Of course, contributors to such an effort would face challenges familiar to any author of information intended to guide patient care, regardless of copyright model. Authors must evaluate the best available evidence—including combined analyses when these provide meaningful conclusions—and must be clear about the quality of that evidence. At the same time, they must bear in mind two considerations that practicing clinicians face daily. First, aggregate results, whether from well-conducted individual studies or meta-analyses, provide only a starting place for assessing the best approach for the individual patient, who brings a unique context of preferences, values, and circumstances. Second, making clinical decisions often requires striking a balance between imperfect knowledge and the consequences, to the extent they can be predicted, of inaction. Where the scientific evidence is not of the highest quality, both writers and readers need somehow to make the best of it.

As a professionally edited, open access medical journal with engaged academic editors from the clinical research community, *PLOS Medicine* seems well positioned to contribute by publishing a limited number of clinical review articles. Our colleagues at *PLOS Computational Biology* have already engaged in an analogous effort, albeit not one focused on patient care [3]. *PLOS Medicine*’s scope would permit us to prioritize topics of particular urgency in settings where resources to access point-of-care information are limited. For the original journal article, the editors would maintain a strong policy on author and reviewer competing interests.

Practical issues remain to be raised and resolved, however. Perhaps the most important concern how to maintain the reliability of a living review. Editors of journals that publish original research can reasonably expect researchers to critique and verify one another’s results both initially and over time, and other interested parties to comment. Facilitating these activities before changes to patient care or policy result is one of the great benefits of open access publishing in medicine. In contrast, clinicians accessing point-of-care summaries, perhaps with limited time or inclination to integrate information from multiple sources themselves, can apply the conclusions of a review to a patient’s living body within seconds of reading the information. Writers and editors of such articles, therefore, must exercise commensurate vigilance in guarding against bias, competing interests, and blurring of the lines between evidence and speculation on ways of applying it. Even in the one-time publication of an article, such vigilance can be demanding; ensuring integrity in a living point-of-care reference would require ongoing processes, which would have to scale with the size of the of collection.

It may be that no currently existing model is perfectly suited to the task. A misplaced decimal point, ambiguous phrasing, or a poorly rendered symbol in a clinical reference has the potential for more immediate harm than in most other kinds of published article. Would an all-volunteer effort like Wikipedia’s be adequate to ensure that the tedious but generally indispensable matter of proofreading, copyediting, and compatibility across software and media platforms will reliably occur across large numbers of summaries? Are the community standards around open re-use, which work so well for original research, optimal to ensure that competing interests excluded from a “seed” clinical review do not come to dominate subsequent versions appearing on other platforms? Will clinicians be comfortable relying on patient care information if authors are not prominently identified or may change without notice? Will authors or publishers of “seed” reviews be comfortable relinquishing control of the “sprouts?”

We believe that these issues are ripe for resolution, provided that interested journals, online information resources, potential authors, and funding agencies are motivated to focus their creative attention on them. While the *PLOS Medicine* Editors welcome the opportunity to participate, we feel that no single journal would serve the spirit or the effectiveness of the enterprise by seeking an exclusive role. For the production of freely available, scrupulously well written, and frequently updated clinical reviews to become a part of the future medical world—and there are global clinical benefits if they do—there will be ample work for many hands and organizations in creating, disseminating, and maintaining them.

Will communities of clinical experts engage in a sustained effort to maintain open access, point-of-care resources at the high level of quality that patient care demands? We hope so, and we encourage those who feel inspired by the potential scope and benefits of such a project to join forces in building it.
